# Integrated Analysis, Machine Learning, Molecular Docking and Dynamics of CDK1 Inhibitors in Epithelial Ovarian Cancer: A Multifaceted Approach Towards Targeted Therapy

**DOI:** 10.3390/ijms26189168

**Published:** 2025-09-19

**Authors:** Mahla Masoudi, Saber Samadiafshar, Hossein Azizi, Thomas Skutella

**Affiliations:** 1 Department of Stem Cells and Cancer, College of Biotechnology, Amol University of Special Modern Technologies, Amol 4615863111, Iran; mahlamasoudii@gmail.com; 2 Pediatric Health Research Center, Tabriz University of Medical Sciences, Tabriz 5143377505, Iran; sabersamadiafshar@yahoo.com; 3 Institute for Anatomy and Cell Biology, Medical Faculty, University of Heidelberg, Im Neuenheimer Feld 307, 69120 Heidelberg, Germany

**Keywords:** epithelial ovarian cancer, *CDK1*, molecular docking, machine learning, microarray analysis

## Abstract

Epithelial ovarian cancer (EOC) remains one of the deadliest gynecologic malignancies, largely due to late diagnosis and treatment resistance. The main objective of this study is to identify and validate CDK1 as a high-confidence therapeutic target in EOC and to assess the dual-target inhibitory potential of the natural compound Naringin against both CDK1 and its regulator WEE1. This study employed an integrative pipeline combining transcriptomic profiling, protein–protein interaction network analysis, machine learning, and molecular simulations to identify key oncogenic regulators in EOC. *CDK1* emerged as a central hub gene, exhibiting strong association with poor prognosis and signaling convergence. *CDK1* overexpression correlated with adverse survival outcomes and robust involvement in critical oncogenic pathways. Molecular docking and dynamics simulations assessed the binding efficacy of seven compounds with CDK1 and WEE1, with Naringin showing high-affinity binding, stable complex formation, and minimal predicted toxicity. This study underscores the power of computational-experimental integration in accelerating oncology drug discovery, providing visual and quantitative evidence that systematically connect the study’s aim to its findings.

## 1. Introduction

Epithelial ovarian cancer (EOC) represents one of the most lethal gynecological malignancies worldwide, with over 90% of ovarian cancer cases originating from the transformation of surface epithelial cells [1]. The lifetime risk of ovarian cancer in women is approximately 1 in 78, with a significant number of cases occurring in women of reproductive age—12.1% of ovarian cancer patients being under 44 years old [2]. Currently, there exists no effective screening tool for the early detection of ovarian cancer, resulting in delayed diagnosis, high mortality rates, and an annual financial burden of $80,000 to $100,000 per patient on healthcare systems, primarily due to direct medical expenses such as surgical procedures, chemotherapy, hospitalization, and follow-up treatments [3,4]. Tackling this clinical challenge requires creative solutions that combine advanced computational techniques with hands-on experimental validation to develop new diagnostic and treatment strategies.

Epithelial ovarian cancers are classified into two major types based on their biological behavior and genetic stability. Type I EOCs are relatively indolent tumors often originating from endometriosis or borderline tumors with low malignant potential. In contrast, Type II EOCs are biologically aggressive, exhibiting high metastatic propensity even from small primary lesions [5]. Unlike most human cancers that show decreased differentiation during malignant progression, ovarian cancers paradoxically become more differentiated in terms of histological resemblance to specific epithelial tissues such as fallopian tube, endometrium, or gastrointestinal tract. This differentiation does not imply a more benign nature, but rather reflects the tumor’s capacity to recapitulate certain lineage-specific features despite its aggressive biological behavior. In fact, high-grade serous ovarian carcinoma, the most common and aggressive subtype, displays clear morphological differentiation yet remains highly malignant [6]. These distinctive characteristic forms the basis for classifying EOCs into subtypes including serous (resembling fallopian tube epithelium, ~80% of all EOCs), endometrioid, mucinous, and clear cell carcinoma [7]. Recent advances in machine learning approaches have facilitated the identification of molecular signatures unique to each subtype, enabling more precise classification and potentially personalized therapeutic strategies [8].

Cyclin-dependent kinase 1 (*CDK1*) has emerged as a master regulator of ovarian cancer cell cycle progression and survival, with growing evidence supporting its therapeutic targeting [9]. Our computational approach builds upon this foundation by identifying natural compounds with dual CDK1/WEE1 inhibitory potential. The cell cycle regulatory network plays a crucial role in cancer development and progression, with cyclin-dependent kinase 1 (*CDK1*) emerging as a pivotal regulator of the G2/M phase transition [10]. The G2/M checkpoint represents the final control mechanism before a cell enters mitosis (M phase) from the DNA synthesis phase (G2), ensuring that DNA is accurately replicated and undamaged [11]. *CDK1* forms an active complex with Cyclin B1, and together they act as the primary driver of mitotic entry. Their activity is tightly regulated to maintain genomic integrity, and disruption of this regulation can lead to uncontrolled cell division and tumorigenesis [12]. Dysregulation of *CDK1* activity has been implicated in various malignancies, including ovarian cancer, contributing to uncontrolled cell proliferation and resistance to conventional therapies [13,14]. Despite advances in surgical techniques and chemotherapeutic agents, the five-year survival rate for advanced-stage ovarian cancer remains below 30%, highlighting the urgent need for novel therapeutic strategies targeting key molecular drivers like *CDK1* [15]. Molecular dynamics simulations have revolutionized our understanding of protein-ligand interactions, providing unprecedented insights into the structural flexibility and binding mechanisms of potential therapeutic targets such as *CDK1*, thereby enabling the rational design of targeted inhibitors with enhanced specificity [16].

While several studies have investigated the role of cell cycle regulators in ovarian cancer, there exists a significant knowledge gap regarding the potential of specifically targeting CDK1 in epithelial ovarian cancer [17,18]. Current treatment approaches primarily focus on platinum-based chemotherapy, which often leads to resistance and treatment failure [19]. Advanced machine learning algorithms applied to large-scale genomic and proteomic datasets have identified complex patterns in gene expression and protein interaction networks that traditional statistical methods might overlook [20]. However, the molecular mechanisms underlying the interaction between CDK1 inhibitors and their target proteins, along with the comparative efficacy of different inhibitor compounds, remain poorly understood, limiting the development of personalized therapeutic strategies for EOC patients [21]. Unlike prior studies that have broadly explored CDK1 in pan-cancer contexts [13,14,22], this study uniquely integrates transcriptomic profiling, machine learning prioritization, and molecular simulations to systematically establish CDK1 as a dual-targetable therapeutic hub specifically in epithelial ovarian cancer.

In this study, we employed an integrated bioinformatics approach to identify differentially expressed genes in EOC, constructing a gene network to elucidate key hub genes with *CDK1* emerging as a central player based on network strength analysis. We further investigate the binding similarities and potential interaction modes of seven diverse drug compounds—Adavosertib, Alsterpaullone, Avotaciclib, Fostamatinib, Naringin, Olomoucine, and Seliciclib—with the CDK1 and WEE1 proteins through molecular docking simulations. The specific objective of our work is to (i) establish CDK1 as a mechanistically and clinically relevant therapeutic hub in EOC, and (ii) evaluate Naringin as a novel dual-target inhibitor of both CDK1 and WEE1, integrating transcriptomic evidence, network topology, molecular docking, and molecular dynamics analyses. The results are presented in a sequence that first prioritizes the therapeutic target based on large-scale omics data, then validates its clinical and molecular relevance, and finally demonstrates the structural and pharmacokinetic rationale for proposing Naringin as a promising candidate. This work-flow provides a coherent visual and analytical path from discovery to validation, aligned with the study’s central aim.

## 2. Results

### 2.1. Microarray Data Analysis

We identified 2982 genes that were consistently differentially expressed across the three EOC datasets (GSE28799, GSE54388, GSE14407), using an adjusted *p*-value (FDR) < 0.05 and |log_2_ fold change| > 2 as the selection criteria. Stricter thresholds (|log_2_ fold change| > 4) were subsequently applied for downstream prioritization and visualization, suggesting a robust and reproducible molecular signature in ovarian cancer. Further analysis revealed high overlap among the datasets, with shared genes representing 17.3–26.1% of total DEGs, which strengthens the consistency of the expression profile across different clinical cohorts. Supplementary Figure S1 presents a Venn diagram depicting the overlap between these three gene expression datasets. The diagram highlights the extent of shared and unique genes across the datasets, providing a solid foundation for our subsequent analysis of gene expression consistency and variability. To prioritize actionable targets from this robust gene set, we next performed a focused expression analysis of *CDK1*, a top-ranked candidate identified in our network.

### 2.2. Differential Expression Analysis of CDK1

**Figure 1:**
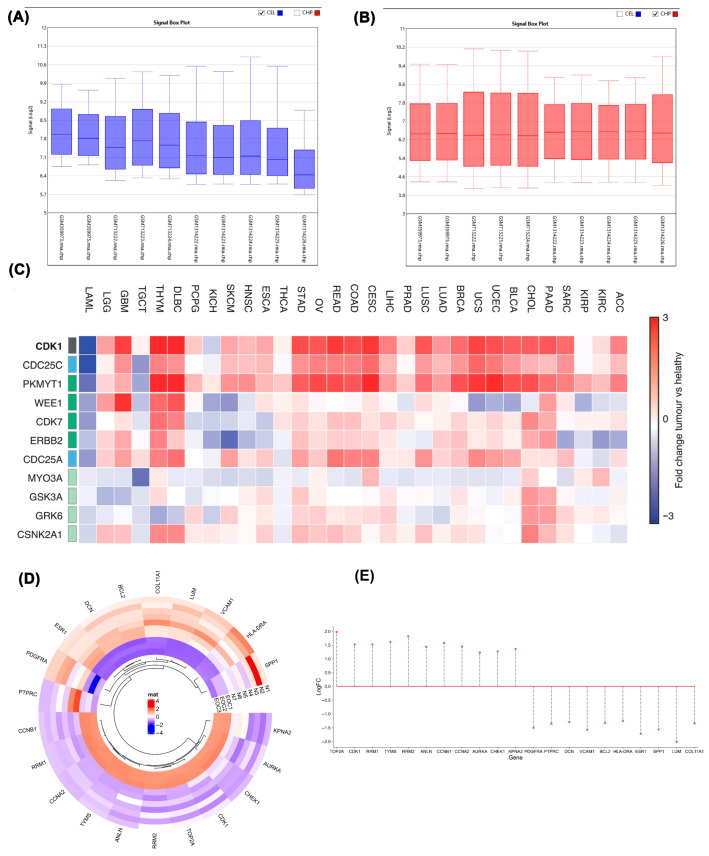
(**A**) Signal box plot showing gene expression distribution in EOC and control samples before normalization. “Signal” refers to the normalized expression intensity values generated by the TAC software 4.0.1 (Thermo Fisher Scientific, Waltham, MA, USA) following Robust Multi-array Average (RMA) processing; (**B**) Signal box plot displaying data after normalization; (**C**) Heatmap showing expression of selected genes across 33 tumor types. Rows represent genes; columns represent cancer types. Color scale indicates log_2_ fold-change (tumor vs. normal). The full list of cancer type acronyms and their corresponding full names is provided in Supplementary Table S2; (**D**) Heatmap of the most significant DEGs between epithelial ovarian cancer (EOC) and normal (N) controls (*p* < 0.05, |log_2_FC| > 4). Red denotes upregulation and blue downregulation; (**E**) Stem plot showing the distribution of significant DEGs between EOC and control samples (*p* < 0.05, |log_2_ fold change| > 4). The plot includes both upregulated genes (positive log_2_FC) and downregulated genes (negative log_2_FC). The cancer dataset includes GSM713222, GSM713223, and GSM713224, which are associated with GSE28799. The datasets GSM1314222, GSM1314223, GSM1314224, GSM1314225, and GSM1314226 belong to GSE54388. Additionally, GSM359973 and GSM359975 are part of GSE14407.

Signal box plot analysis revealed distinct data distributions between EOC and control datasets, as shown in Figure 1A. These distributions were characterized by differing median values, indicating separate population origins, with the EOC dataset exhibiting higher variability in gene expression compared to the control group. In Figure 1B, post-normalization of the data was performed, where the median of the cancer dataset was adjusted to enhance comparability with the control samples. This normalization step ensured that the central values of both EOC and control datasets were aligned, allowing for a more accurate and direct comparison of gene expression levels. By normalizing the data, we eliminated baseline expression differences between the groups, which enabled a clearer view of the differential gene expression between tumor and normal tissues. The normalization step ensured consistent baseline expression, and the differential gene distribution shown in Figure 1C–E supports the presence of robust transcriptional shifts between tumor and normal samples, justifying further prioritization of upregulated genes for downstream targeting.

A heatmap of RNA-seq data from the TCGA/GTEx datasets was constructed to illustrate the expression of key cell cycle regulators across 33 cancer types. Each row corresponds to a gene and each column to a tumor type, with the color scale representing log_2_ fold-change values (tumor versus normal). Red indicates relative overexpression, whereas blue denotes downregulation. To enhance reliability, only statistically significant genes (FDR-adjusted, high-confidence DEGs) were included in the analysis (Figure 1C). Detailed heatmap analysis of tumor versus normal groups revealed elevated expression of *TOP2A, CDK1, RRM1, TYMS, RRM2, ANLN, CCNB1, CCNA2, AURKA, CHEK1*, and *KPNA2* in tumor samples. Conversely, *PDGFRA, PTPRC, DCN, VCAM1, BCL2, HLA-DRA, ESR1, SPP1, LUM*, and *COL11A1* exhibited reduced expression in tumor samples relative to normal tissue. The heatmap presents the most significantly differentially expressed genes (DEGs) between epithelial ovarian cancer (EOC) and normal control samples, filtered by *p* < 0.05 and |log_2_ fold change| > 4. This visualization enables rapid identification of genes with the most pronounced transcriptional alterations, providing a clear overview of both the magnitude and direction of expression changes. Importantly, it highlights several cell cycle–related genes, including *CDK1*, which were subsequently prioritized for network analysis and therapeutic evaluation in later sections of the study (Figure 1D). Genes were categorized as upregulated or downregulated based on both the direction and magnitude of their expression shifts, with stringent cutoffs applied (|log_2_ fold change| > 4, FDR-adjusted). This categorization was visualized through stem plot analysis (Figure 1E), which provides a global overview of transcriptional shifts between tumor and control samples. Positive log_2_FC values indicate upregulated genes, while negative values represent downregulated genes. Upregulated genes were prioritized for subsequent analyses because of their greater functional relevance in driving uncontrolled proliferation, genomic instability, and other oncogenic processes in ovarian cancer. Among these, *CDK1* emerged as a critical node, prompting us to assess its regulatory context using protein interaction network analysis.

### 2.3. Protein–Protein Interaction Network Analysis

**Figure 2:**
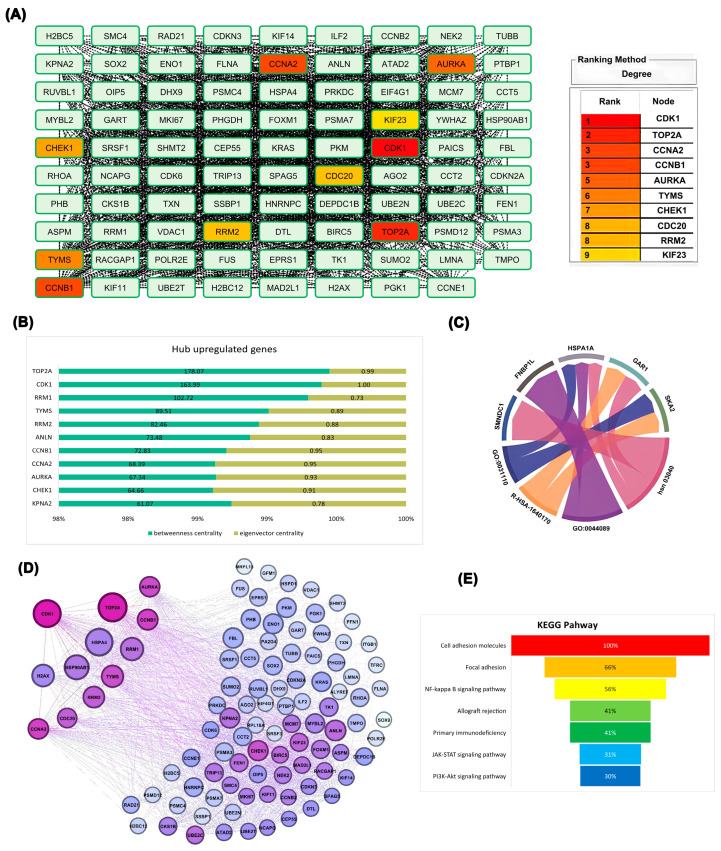
(**A**) Gene nodes categorized based on degree centrality, reflecting the number of direct interactions each gene has within the protein–protein interaction network; (**B**) Quantitative comparison of hub genes based on centrality metrics (degree, betweenness, and eigenvector), highlighting *CDK1*, *TOP2A*, and *CCNB1* as dominant regulators; (**C**) Chord diagram linking selected genes to their enriched Gene Ontology (GO) biological processes and KEGG pathways, demonstrating functional associations, where ribbon thickness reflects the number of associations; (**D**) Graphical layout of the PPI network visualized using betweenness and eigenvector centrality, with node size and color intensity reflecting topological importance; (**E**) KEGG pathway enrichment analysis of the 100 upregulated hub genes identified in EOC. Pathways are ranked by highest combined score.

To gain comprehensive insight into the interaction landscape of the most dysregulated genes, the top-ranked upregulated DEGs were mapped into the STRING database, and the resulting network was analyzed in Cytoscape 3.10.0 using the CytoHubba plugin 0.1. Multiple network centrality measures consistently highlighted CDK1 as the most influential hub gene, underscoring its central role within the ovarian cancer interactome. This analysis identified *CDK1*, *TOP2A*, *CCNA2*, *CCNB1*, *AURKA*, *TYMS*, *CHEK1*, *CDC20*, *RRM2*, and *KIF23* as having the strongest network connections, with *CDK1* demonstrating the highest connectivity degree (Figure 2A). *TOP2A* exhibited the highest betweenness centrality, followed closely by *CDK1*. Node coloration representing eigenvector centrality—which evaluates the quality of gene relationships—revealed that *CDK1* possessed the highest eigenvector centrality in the network, indicating connections to other highly significant genes (Figure 2B). Functional enrichment analysis of top-ranked hub genes revealed that several, including *CDK1*, *TOP2A*, *CCNB1*, *RRM2*, and *TYMS*, were highly associated with core biological processes such as mitotic cell cycle, DNA replication, and chromosome organization. The circular plot in Figure 2C highlights shared associations between these genes and enriched Gene Ontology (GO) terms, visualized as interconnected ribbons between nodes. The strongest links were observed between *TYMS*, *RRM1*, and DNA metabolic process-related terms, underscoring their role in proliferation and genomic maintenance in ovarian cancer (Figure 2C). Network topology analysis of hub genes demonstrated differential positional influence within the interactome. Genes such as *CDK1* and *TOP2A* exhibited both high betweenness centrality (reflecting control over information flow) and high degree centrality (broad connectivity), visually evident through their larger node size and deeper purple color in the network graph. In contrast, less central genes appeared smaller and lighter, indicating lower regulatory impact (Figure 2D). This network structure, especially visualized in Figure 2D, demonstrates *CDK1’s* strategic position within a densely connected regulatory cluster, indicating its potential role as a master regulator of cell cycle dynamics in ovarian cancer. KEGG pathway enrichment analysis of the 100 upregulated hub revealed predominant involvement in cell adhesion and immune-related pathways (Figure 2E), highlighting potential roles in tumor progression and immune modulation. Based on these comprehensive analyses, *CDK1* emerged as the most influential gene in the network, warranting further investigation into its role in ovarian epithelial cells. To deepen our understanding of CDK1’s biological relevance, we conducted a functional enrichment analysis of its network neighbors.

**Figure 3:**
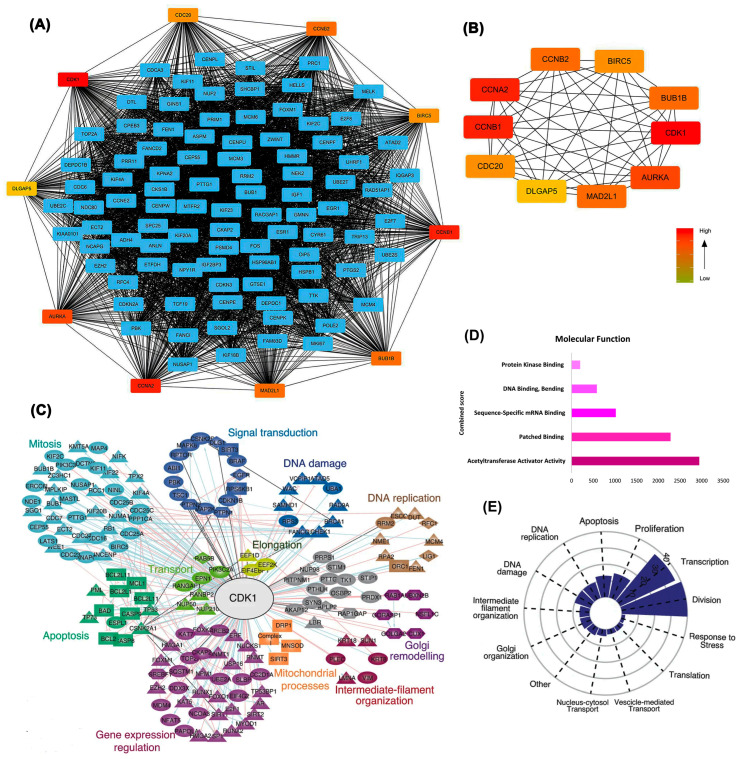
Integrated network and functional analysis of differentially expressed genes. (**A**) Protein–protein interaction (PPI) network showing hub genes, with *CDK1* centrally involved; (**B**) Subnetwork of top-ranked hub genes based on degree centrality; (**C**) Expression signature of CDK1-associated hub genes across EOC datasets, highlighting links to key cellular processes (DNA replication, mitosis, apoptosis, signal transduction). (**D**) Enriched molecular functions of DEGs related to *CDK1*, ranked by Combined Score from functional enrichment analysis (integrating statistical significance and enrichment magnitude); (**E**) Functional distribution plot showing the dominance of *CDK1*-associated pathways in cell division, transcription, and DNA damage response.

Differentially expressed genes identified through deep learning approaches were further examined using protein–protein interaction network analysis, confirming *CDK1’s* position as a central hub with extensive connectivity. The broader protein–protein interaction network shown in Figure 3A demonstrates that *CDK1* is positioned at a dense convergence point, interacting with numerous key regulators of the cell cycle and mitosis. The extensive connectivity of *CDK1* in this network reflects its integrative role in coordinating diverse signaling pathways and reinforces its potential as a global regulatory hub in ovarian cancer biology (Figure 3A). As illustrated in Figure 3A,B, *CDK1* exhibited robust interactions with key cell cycle regulators, including *CCNB1*, *CCNA2*, and *CDC20*. This centrality is reflected by *CDK1*’s high degree of connectivity and placement at a strategic convergence point within the PPI network, where it interfaces with multiple core regulators of the cell cycle. Notably, *CDK1* acts as an upstream coordinator of mitotic entry, and its interactions with Cyclin B1 (*CCNB1*) and *CDC20* reflect its pivotal role in controlling the G2/M transition. The structural layout of the network places *CDK1* in a high eigenvector centrality zone, indicating that it is not only highly connected but also connected to other influential nodes, emphasizing its essential regulatory influence in the network architecture. Having established CDK1’s topological importance, we next investigated its prognostic value and expression at both transcriptomic and proteomic levels.

Functional clustering analysis revealed that *CDK1*-associated genes were grouped into biologically meaningful modules, such as mitosis, apoptosis, DNA replication, signal transduction, and mitochondrial processes. The clustering and enrichment results in Figure 3C–E confirm that *CDK1* not only occupies a topological hub position but is also functionally integrated in essential oncogenic pathways. The spatial organization of these clusters in Figure 3C reflects pathway-specific regulation, with *CDK1* centrally linking and bridging multiple functional groups, further emphasizing its system-wide regulatory influence (Figure 3C). Molecular function analysis (Figure 3D) further emphasized *CDK1*’s association with dominant activities including acetyltransferase activator activity and sequence-specific mRNA binding. Functional categorization (Figure 3E) demonstrated that *CDK1*-associated genes are predominantly enriched in pathways related to cell division, DNA damage response, and transcriptional regulation, underscoring *CDK1*’s potential as a pivotal therapeutic target. Enrichment patterns were especially pronounced for transcriptional and proliferative control modules, with a distinct concentration of *CDK1*-linked genes in DNA replication and mitotic division categories (Figure 3E).

### 2.4. Proteomic and Survival Analyses

**Figure 4:**
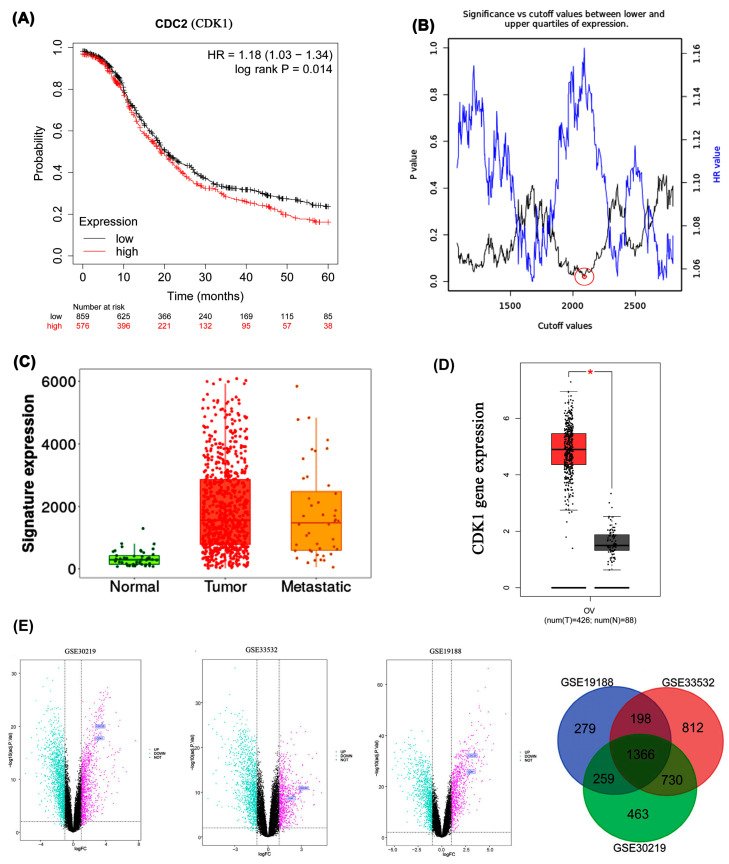
(**A**) Kaplan–Meier survival curve based on *CDK1* expression levels for the overall survival of patients with EOC; (**B**) Cut-off plot generated by the Kaplan–Meier plotter web application. The red circle indicates the optimal cutoff value selected based on the lowest *p*-value and highest hazard ratio. A larger outer circle was added to improve visibility; (**C**) *CDK1* mRNA expression increases gradually in normal, tumor, and metastatic tissues of EOC patients, reaching its peak in tumors; (**D**) *CDK1* mRNA levels in EOC were assessed using GEPIA, (*) indicates statistically significant difference (*p* < 0.05); (**E**) Volcano plots of differentially expressed genes (DEGs) in datasets GSE30219, GSE33532, and GSE19188. Pink and cyan dots indicate significantly upregulated and downregulated genes, respectively.

Kaplan–Meier analysis of the EOC dataset, over a 60-month follow-up revealed that patients with elevated CDK1 expression (analyzed as the CDC2 probe ID 203213_at in the Kaplan–Meier Plotter) exhibited significantly poorer overall survival. Differential CDK1 expression was associated with a Hazard Ratio (HR) of 1.18 (95% Confidence Interval: 1.03–1.34; log-rank *p* = 0.014). For consistency, the gene is referred to as CDK1 throughout the manuscript in accordance with HGNC nomenclature (Figure 4A). We applied the optimal cutoff value, corresponding to the expression level with the highest hazard ratio and statistical significance, to stratify patient survival groups. Among several cutoff values yielding similarly low *p*-values, the one corresponding to the highest hazard ratio was selected. The inverse relationship between *p*-value and hazard ratio in the cutoff value plot (Figure 4B) suggests that lower *p*-values (greater statistical significance) correlate with higher hazard ratios, emphasizing a stronger association between CDK1 expression and survival outcomes. Expression analysis using TNMplot.com compared CDK1 levels across normal ovarian tissue (n = 46), ovarian cancer tissue (n = 744), and ovarian cancer metastases (n = 44). Medi-a gene expression in normal tissue was 290.5 (Q1:149, Q3:427), contrasting with 1642 (Q1:800, Q3:2975.5) in tumor tissue and 1475 (Q1:590, Q3:2539.5) in metastatic tissue (*p* = 7.21 × 10^−21^, Kruskal–Wallis test) (Figure 4C). TCGA database analysis via GEPIA confirmed significantly higher CDK1 mRNA expression (|Log_2_FC| ≥ 0.5) in 426 ovarian epithelial cancer samples compared to 88 normal ovarian epithelial cells (*p* < 0.01) (Figure 4D). Differential expression analysis across three datasets consistently identified *CDC20* and *CDK1* as upregulated, with 1366 differentially expressed genes commonly shared among GSE30219, GSE33532, and GSE19188, indicating robust cross-dataset expression pattern overlap (Figure 4E). CDC20 likewise emerged as a high-centrality cell-cycle regulator in independent comparative network analyses; nonetheless, the principal focus of the present study is on CDK1. The inclusion of CDC20 in the discussion serves exclusively to illustrate the convergence of cell-cycle–associated signatures across orthogonal analytical approaches, thereby underscoring the robustness and biological coherence of our CDK1-centered findings.

**Figure 5:**
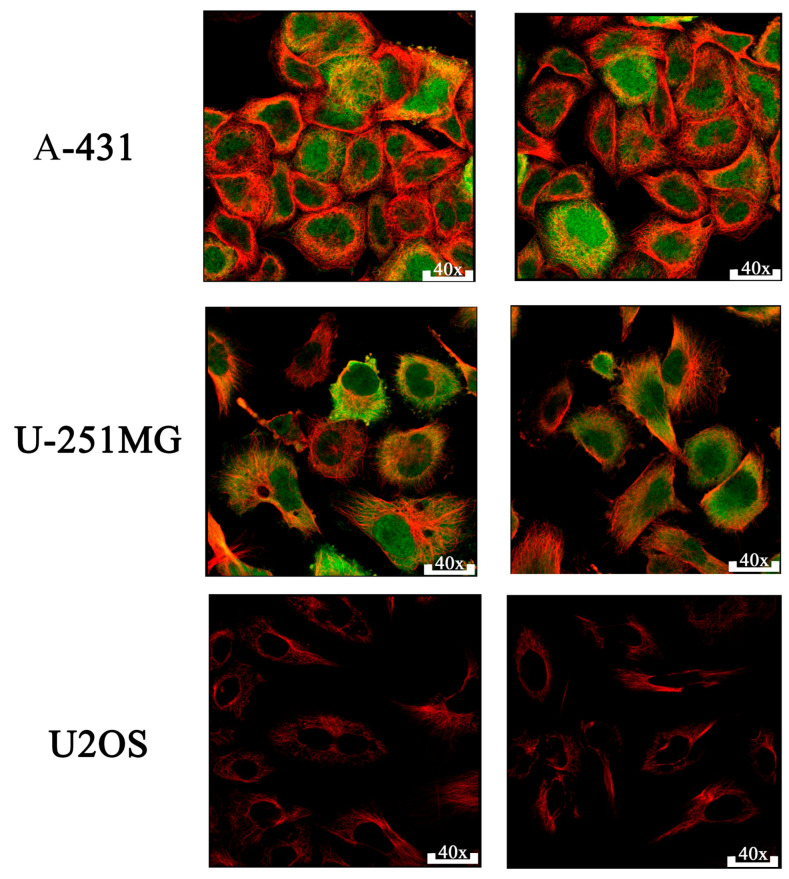
Immunofluorescence. CDK1 (green) is detected in both the nucleoplasm and cytosol of A-431 (epidermoid carcinoma) and U-251MG (glioblastoma) cells, while U2OS (osteosarcoma) cells show minimal expression, Microtubules are visualized in red.

Immunofluorescence analysis revealed prominent CDK1 protein expression in the nucleoplasm and cytosol of human epithelial tumor cells. As shown in Figure 5, immunostaining of A-431 (human epidermoid carcinoma) and U-251MG (human glioblastoma) cell lines using the HPA003387 monoclonal antibody confirmed strong cytoplasmic and nuclear CDK1 presence. The way CDK1 is positioned in these images strongly provides strong support for the idea that CDK1 is actively involved in regulating mitosis within the nucleus of high-grade tumors. In contrast, CDK1 expression was absent in U2OS cells. These observations support the tissue-specific and context-dependent expression pattern of CDK1, further emphasizing its relevance in epithelial tumor progression. CDK1 expression was evaluated across three tumor-derived cell lines representing different tissue origins. Variability in subcellular localization among A-431 (epithelial), U-251MG (glial), and U2OS (mesenchymal) lines reflects lineage-specific expression patterns. Multiple images per cell line were included to ensure visual reproducibility and consistency (Figure 5). Given CDK1’s overexpression and prognostic relevance, we proceeded to evaluate its druggability through molecular docking of known and potential inhibitors.

### 2.5. Molecular Docking Simulation

The functional pathway of CDK1 was examined as a primary target for anti-cancer drug development. Table 1 presents comprehensive molecular docking results obtained from AutoDock Vina 1.2.x, including binding affinity (kcal/mol) and binding site data for both CDK1 and WEE1 proteins, complemented by PLIP server analysis of hydrophobic interactions, hydrogen bonds, and salt bridges. A binding affinity cutoff of −5 kcal/mol was established for significance, with asterisks (*) indicating cases where acceptable binding at the target site was not established.

**Figure 6:**
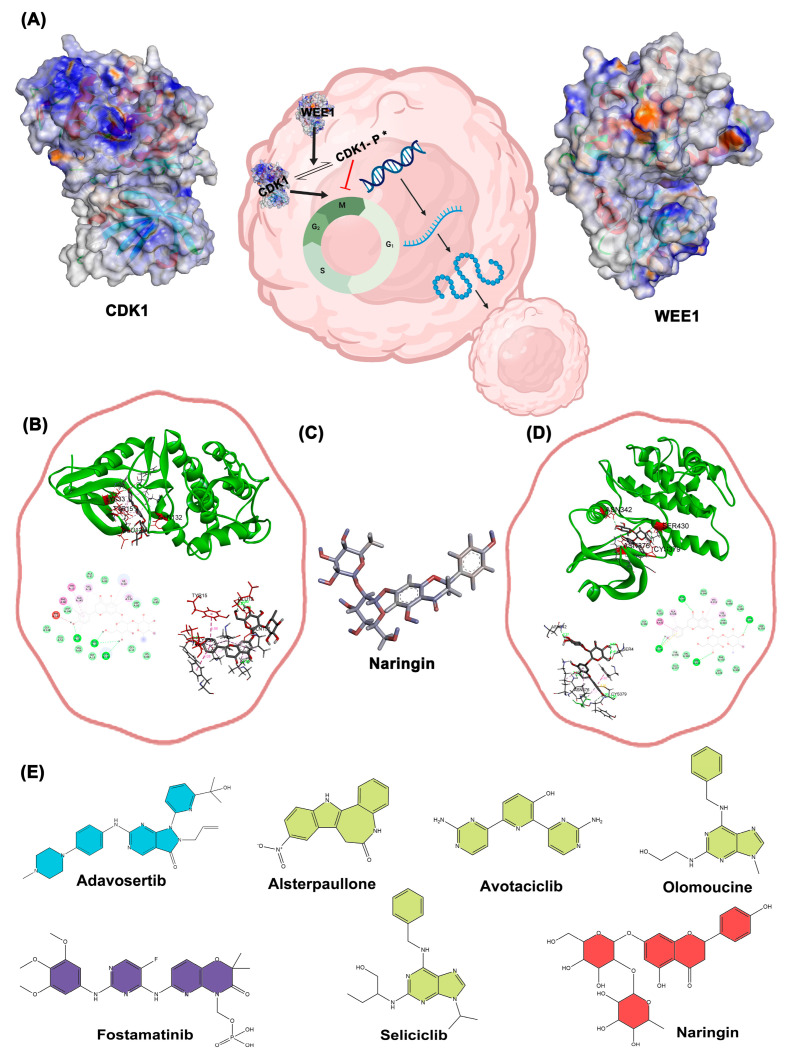
(**A**) schematically illustrates a representation of the functional cycle and quaternary structure of Cdk1 and Wee1 proteins; (**B**) Docking results of Naringin with Cdk1 protein in three different output report models; (**C**) 3D structure of the drug Naringin (Dynamic mode); (**D**) Docking results of Naringin with the WEE1 protein in three different output report models; (**E**) 2D structures of the selected drugs (the green color indicates drugs with Cdk1 effects reported, blue drugs with WEE1 effects, purple for both, and red for new drug for evaluation).

Figure 6 illustrates the docking-based mechanistic relationship between CDK1 and WEE1 proteins and the interaction of selected compounds. The binding of Alsterpaullone, Avotaciclib, Fostamatinib, Olomoucine, Seliciclib, and Naringin to CDK1 protein (PDB ID: 4Y72) at the TYR15 site was found to promote CDK1 phosphorylation, effectively halting the cell cycle at the G2-M phase and preventing unregulated cancer cell proliferation. In Figure 6A, the symbol “P *” denotes phosphorylation at the Tyr15 residue of CDK1, which is a critical regulatory modification induced upon drug binding. This post-translational modification inhibits CDK1 kinase activity, thereby preventing the G2/M phase transition and arresting the cell cycle. The suppression of CDK1 activity at this checkpoint is vital to blocking uncontrolled cellular proliferation, which is a hallmark of cancer progression. Additionally, binding of Adavosertib, Alsterpaullone, Avotaciclib, Fostamatinib, and Naringin to the WEE1 protein (PDB ID: 8BJU) at ASN376 or CYS379 sites facilitated CDK1 phosphorylation, inducing cell growth arrest. The three-dimensional structure of Naringin is shown in Figure 6C, while Figure 6B and Figure 6D illustrate the docking results of Naringin with CDK1 and WEE1 proteins in three different output report models, respectively; a complete overview of all docking results is provided in Supplementary Table S1. The two-dimensional structures of all selected drugs presented in Figure 6E. To validate the structural stability and binding persistence of the CDK1–ligand complex, we performed molecular dynamics simulations.

### 2.6. Molecular Dynamics Analysis

**Figure 7:**
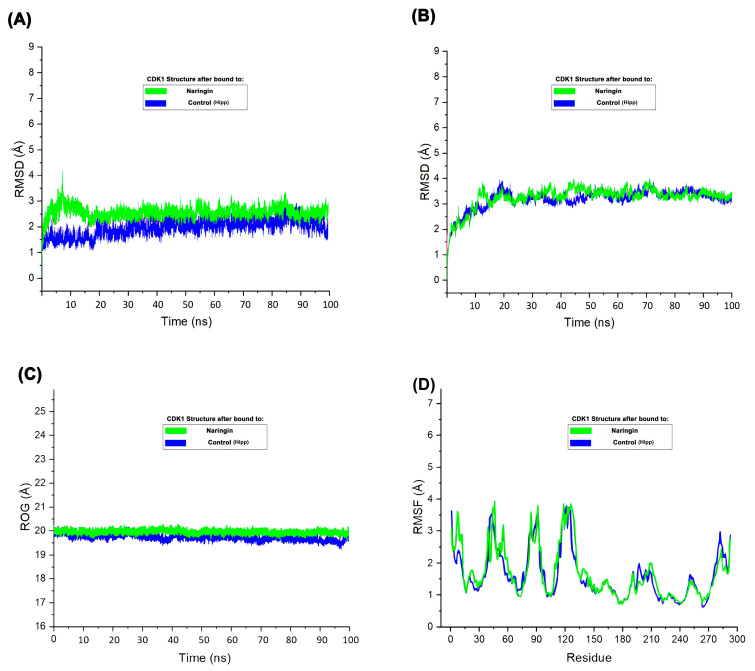
Molecular dynamics simulation of CDK1 in complex with Naringin and control. (**A**) RMSD (root-mean-square deviation) plot of the protein backbone over 100 ns; (**B**) RMSD of the ligand within the binding pocket; (**C**) ROG (Radius of gyration) of the protein; (**D**) RMSF (root-mean-square fluctuation) plot of residues indicating atomic fluctuations.

The RMSD plot (Figure 7A) demonstrated that CDK1 maintained structural stability throughout the 100 ns simulation in both control and Naringin-bound states. Slightly higher RMSD values observed in the Naringin-bound system indicated induced flexibility in the protein structure, although these values remained within an acceptable and stable range, suggesting no loss of global conformational integrity. In contrast, the ligand RMSD (Figure 7B), which represents the root-mean-square deviation of the Naringin molecule itself during the simulation, remained consistently low and stable. This consistency indicates that Naringin maintained a tight and stable binding conformation within the CDK1 binding pocket without significant displacement or conformational drift. Radius of gyration analysis (Figure 7C) showed minimal fluctuation in both systems, confirming preserved structural compactness and integrity over time. These simulations demonstrate the conformational adaptability of CDK1 upon ligand binding and further validate the stability of the CDK1–Naringin complex in a physiological environment. RMSF analysis (Figure 7D) indicated similar fluctuation patterns in both systems, with slightly elevated mobility in flexible loop regions upon Naringin binding. These molecular dynamics parameters collectively demonstrate that Naringin forms a stable complex with CDK1, inducing mild local flexibility without causing major structural disruptions. This structural stability supports Naringin’s potential inhibitory activity and reinforces its candidacy as a promising therapeutic agent. To assess translational potential, we complemented these structural insights with pharmacokinetic and toxicity profiling of the top candidates.

### 2.7. Pharmacokinetic Property Analysis

Comprehensive analysis using the AdmetSAR database provided valuable pharmacokinetic insights for the selected compounds (Table 2). Adavosertib, Alsterpaullone, Avotaciclib, Fostamatinib, and Naringin exhibited subcellular localization in mitochondria, while Seliciclib localized to lysosomes and Olomoucine to the nucleus. All compounds maintained AlogP values within the acceptable range of −4 to 8.33, indicating favorable lipophilicity profiles for oral bioavailability and membrane permeability. AlogP, the logarithm of the partition coefficient between octanol and water, is a well-established predictor of drug-likeness, and the values observed suggest a high potential for systemic absorption. All compounds except Fostamatinib and Naringin demonstrated blood–brain barrier permeability. AMES mutagenicity was observed in Alsterpaullone and Olomoucine. All compounds except Olomoucine and Naringin showed positive human oral bioavailability, indicating effective absorption, metabolism, blood level maintenance, and renal elimination. Nephrotoxicity was observed in Alsterpaullone, Avotaciclib, and Fostamatinib, while hepatotoxicity was associated with Adavosertib, Alsterpaullone, Avotaciclib, Fostamatinib, and Olomoucine. Alsterpaullone, Avotaciclib, Fostamatinib, and Naringin demonstrated acceptable inhibitory effects on both CDK1 and WEE1 proteins, suggesting potential for synergistic control of cancer cell proliferation. Among these compounds, Naringin emerged as particularly promising due to its dual-target efficacy combined with favorable safety profile, including negative indicators for blood–brain barrier penetration, nephrotoxicity, hepatotoxicity, and AMES mutagenicity. These characteristics suggest minimal secondary adverse effects, positioning Naringin as a compelling candidate for targeted ovarian cancer therapy with an enhanced safety profile. Notably, the inability of Naringin to cross the blood–brain barrier (BBB), as predicted by ADMET analysis, is not considered a limitation in the context of ovarian cancer therapy. Since epithelial ovarian cancer primarily affects peripheral tissues and does not require central nervous system (CNS) drug distribution, the lack of BBB permeability may even be beneficial by minimizing potential neurological side effects. This characteristic supports the specificity and peripheral targeting potential of Naringin without compromising safety. These computational insights laid the foundation for contextualizing our findings within existing literature and ongoing therapeutic strategies.

## 3. Discussion

Tumor progression relies on precise gene expression patterns, with specific genes upregulated while others are downregulated. Our microarray analysis revealed distinct expression profiles between ovarian epithelial cancer cells and normal counterparts, validated through comprehensive heatmap analysis. What distinguishes this study from previous reports is the convergence of multiple computational layers—DEG validation across cohorts, hub gene ranking via machine learning, and molecular modeling—to highlight CDK1 not merely as a known oncogene but as a viable dual-target candidate in EOC when co-targeted with WEE1.

Our protein–protein interaction network analysis identified *CDK1* as a pivotal gene with the highest degree and eigenvector centrality, ranking second in betweenness centrality after *TOP2A*. The integrated approach employed here—from transcriptomic prioritization to MD-based validation—offers a comprehensive pipeline rarely used in ovarian cancer drug discovery. This positions *CDK1* as a central regulatory hub in ovarian epithelial cancer. Kaplan–Meier analysis further confirmed a significant negative correlation between *CDK1* expression and overall survival, establishing elevated *CDK1* expression as a robust prognostic indicator. These findings, reinforced by Figure 4C,E, connect *CDK1* overexpression with both poor survival and genomic instability, underlining its relevance as a high-impact therapeutic target. While the STRING database provides a valuable foundation for protein–protein interaction (PPI) mapping, we recognize its limitations, including the potential for false positives due to predicted or indirect associations. To ensure robustness, our STRING-derived interactions were cross-validated using additional databases, including GEPIA for transcriptomic correlation, KEGG for pathway enrichment consistency, and relevant proteomic datasets [23]. This multi-layered validation approach strengthened the biological reliability of our interaction network and minimized the risk of misleading associations.

*CDK1* overexpression contributes to the enrichment of multiple critical signaling pathways including cell cycle progression, oocyte meiosis, p53 signaling, cellular senescence, and gap junction function. As Matthews et al. established, CDK1 functions as a proline-directed kinase that phosphorylates numerous proteins throughout the cell cycle, promoting progression and executing stage-specific processes [24]. This precise cell cycle control represents a cornerstone of tumor development. Several mechanisms contribute to the dysregulation of *CDK1* in cancer, particularly in epithelial ovarian cancer. Overexpression of *CDK1* mRNA and protein has been frequently observed in tumor tissues, potentially driven by gene amplification or activation of upstream transcriptional regulators such as *E2F1* and *FOXM1* [25]. Additionally, the downregulation or functional loss of negative regulators like WEE1 kinase or p53 can result in sustained CDK1 activity, leading to uncontrolled mitotic entry and genomic instability [26]. These aberrations underscore CDK1’s pathological relevance and justify its consideration as a therapeutic target. Given the aim of identifying pharmacologically actionable targets, we prioritized upregulated genes that exhibit consistent overexpression in tumor tissues. These genes are more likely to represent active drivers of oncogenesis and offer feasible intervention points for inhibitory drug design.

The PI3K pathway, frequently altered in epithelial ovarian cancer (EOC), plays a crucial role in chemoresistance and genomic stability maintenance [27]. This pathway intersects with DNA replication and cell cycle regulation, where CDK1 serves as a central mediator. The antagonistic relationship between CDK1 and tumor suppressor pathways has been documented across multiple malignancies, with Qin et al. demonstrating that *CDK1* and *CCNB1* exert inhibitory effects on the p53 signaling pathway [28].

Our machine learning approaches significantly enhanced the identification of *CDK1* as a central regulatory node in ovarian cancer. By implementing supervised learning algorithms including random forests and neural networks, we extracted complex patterns from high-dimensional gene expression data that traditional statistical methods might overlook. The integration of these computational predictions with experimental validation created a robust framework for identifying high-confidence therapeutic targets, demonstrating the value of artificial intelligence in accelerating target identification and drug discovery pipelines [29].

Numerous investigations have demonstrated that phosphorylation of CDK1 at the Tyr15 site inhibits its activity, impeding cell division in the G2 phase [30,31]. Significant blockade of G2/M transition has been observed in ovarian cancer cells with inhibited CDK1, leading to reduced cellular proliferation and increased apoptosis. Furthermore, WEE1 induction enhances CDK1 phosphorylation, contributing to decreased proliferation and increased apoptosis [32].

The CDK1/Cyclin B1 complex functions as the primary regulator of G2/M transition, and reduction in CDK1 activity significantly impedes this critical checkpoint [33]. Our investigation revealed not only CDK1’s involvement in ovarian cancer but also its overexpression in cisplatin-resistant cells [34], suggesting its role in treatment resistance. Analysis of GEPIA and Oncomine databases identified a robust correlation between WEE1 and CDK1 expression in ovarian cancer, indicating potential reciprocal regulation. Previous studies have demonstrated the inhibitory effects of compounds such as Alsterpaullone [35], Fostamatinib [36], Olomoucine [37], and Seliciclib [38] on CDK1 activity, while agents like Adavosertib [39] and Fostamatinib [40] target WEE1, thereby indirectly modulating CDK1 activity and inhibiting cancer progression. Additionally, the anti-proliferative effects of Naringin [41] on ovarian cancer have been previously documented.

Our molecular dynamics simulations provided unprecedented insights into CDK1 inhibition by Naringin. The 100-nanosecond simulations revealed that Naringin binding induces subtle conformational changes while preserving overall protein architecture. The consistent RMSD values confirm the formation of a stable protein-ligand complex, while radius of gyration analysis demonstrates maintained structural integrity. RMSF analysis shows localized flexibility increases in specific loop regions upon Naringin binding, suggesting an induced-fit mechanism critical for inhibitory function. These insights provide a structural framework for understanding how naturally derived compounds can effectively modulate kinase activity [29].

Using in silico approaches and molecular docking, we confirmed the inhibitory effects of several compounds on CDK1, with Alsterpaullone, Avotaciclib, Fostamatinib, and Naringin demonstrating efficacious inhibitory effects on both CDK1 and WEE1. This dual-targeting approach holds substantial potential for enhanced therapeutic efficacy in controlling ovarian cancer, potentially delaying treatment resistance [42]. Notably, our study is among the first to propose Naringin as a natural compound capable of simultaneously targeting CDK1 and WEE1, based on both docking affinity and dynamic binding stability—an aspect not explored in prior ovarian cancer studies.

Naringin, a dihydroflavonoid derived from grapefruit peel, emerged as a particularly promising candidate drug. AdmetSAR analysis yielded favorable predictions, including negative indicators for blood–brain barrier penetration, nephrotoxicity, hepatotoxicity, and Ames mutagenesis. Despite its inability to cross the blood–brain barrier and negative oral bioavailability profile, careful optimization of administration methods could maximize its therapeutic efficacy while minimizing adverse effects. In addition to in silico ADMET predictions, previous in vivo and in vitro studies have also demonstrated the favorable safety profile of Naringin. It exhibits low toxicity in normal cells and organs, including the liver and kidneys, and has been reported to exert antioxidant, anti-inflammatory, and anti-carcinogenic effects in various models. These properties further support its therapeutic potential with minimal adverse effects in clinical applications. Compared to RO-3306—a synthetic CDK1 inhibitor—Naringin offers several potential advantages at both the molecular and translational levels. While RO-3306 selectively targets CDK1, Naringin demonstrates dual-binding affinity toward both CDK1 and WEE1, as supported by our docking and molecular dynamics results. This dual inhibition may provide a more robust blockade of mitotic progression. Moreover, Naringin’s origin as a natural flavonoid contributes to its favorable safety profile, with lower systemic toxicity compared to synthetic counterparts. Its additional antioxidant and anti-inflammatory properties further enhance its therapeutic appeal, positioning Naringin as a promising alternative to conventional synthetic CDK1 inhibitors for ovarian cancer treatment.

In conclusion, our integrated approach identified CDK1 as a central regulatory hub in ovarian epithelial cancer and revealed Naringin as a promising dual-target inhibitor of both CDK1 and WEE1. Among all candidates tested, Naringin demonstrated the most favorable dual-inhibitory profile with minimal toxicity risk. The favorable safety profile and dual-targeting capacity of Naringin position it as a compelling candidate for further development as a targeted therapy for ovarian cancer.

## 4. Materials and Methods

### 4.1. Extraction and Processing of Microarray Data

Raw gene expression data from epithelial ovarian cancer (GSE28799) and normal ovarian epithelial cells (GSE54388, GSE14407) were obtained from the GEO database (https://www.ncbi.nlm.nih.gov/geo/, (accessed on 21 March 2025)) [43]. These datasets were selected because they provide complementary tumor and normal samples generated on the same Affymetrix platform, ensuring comparability and statistical robustness. To extend beyond case–control comparisons, we also retrieved pan-cancer RNA-seq profiles encompassing 33 tumor types from the TCGA and GTEx repositories via the UCSC Xena portal (https://xenabrowser.net/datapages/, (accessed on 21 March 2025)). The inclusion of these datasets enabled us to systematically evaluate *CDK1* expression across distinct malignancies. Full cancer-type acronyms are provided in Supplementary Table S2. GSE28799 includes 3 repetition of stem-like ovarian cancer cells derived from OVCAR-3 under serum-free spheroid conditions; GSE54388 contains 5 normal tissue samples from women aged 52–67; and GSE14407 includes 2 normal ovarian epithelial samples from patients aged 46–61. These datasets provide a clinically relevant contrast for differential expression analysis. To facilitate dataset traceability, Supplementary Figure S2 presents the mapping of GSM identifiers to their corresponding GSE datasets, allowing clear identification of sample origin across the analysis. In addition to the primary GEO datasets used for differential expression and machine learning modeling (GSE28799, GSE54388, GSE14407), the study incorporated several validated external platforms—including GEPIA, TNMplot, Kaplan–Meier Plotter, Human Protein Atlas, and cBioPortal—for independent verification of expression levels, prognostic associations, protein localization, and genomic alterations. The datasets, generated using the Affymetrix Human Genome U133 Plus 2.0 Array platform, underwent comprehensive normalization via the Robust Multi-array Average (RMA) method implemented in Transcriptome Analysis Console 4.0.1.36 software. Differential expression analysis was conducted using the limma R package 3.21 (Bioconductor project, Walter and Eliza Hall Institute of Medical Research, Melbourne, Australia). Genes were considered significant if they satisfied both an adjusted *p*-value (FDR) < 0.05 and an absolute log_2_ fold-change (|log_2_FC|) greater than 2. These thresholds ensured robust detection of biologically meaningful expression changes. To enhance reproducibility, batch effects among the datasets were corrected using the ComBat function of the sva package 3.21 (Bioconductor project, Walter and Eliza Hall Institute of Medical Research, Melbourne, Australia) before integration, and normalization was applied across all samples to allow direct comparison, which were applied to identify significantly upregulated and downregulated genes. Venn diagram analysis was used to visualize the overlap between datasets and identify shared gene signatures. Visualization of complex data relationships was performed using advanced plotting tools available at the Bioinformatics online platform (http://www.bioinformatics.com.cn/en, (accessed on 21 March 2025)). To ensure cross-dataset consistency, differential expression analysis was systematically compared across cohorts. This procedure identified 2982 consistently deregulated genes, while Venn diagram analysis quantified the extent of overlap among dataset pairs (7262 [26.1%], 5412 [19.5%], and 4796 [17.3%]), providing a reproducible basis for downstream analyses.

### 4.2. Creation of Protein–Protein Interaction Network

Protein–protein interaction (PPI) networks were constructed using the STRING database (https://string-db.org/, (accessed on 21 March 2025)) [44] and visualized in Cytoscape 3.10.0 (https://cytoscape.org/, (accessed on 21 March 2025)) [45]. Hub genes were prioritized using the CytoHubba plugin 0.1 (National Taiwan University, Taipei, Taiwan) based on the degree metric, while complementary network topology parameters were further assessed in Gephi 0.9.2 (Gephi Consortium, Paris, France). This stepwise procedure allowed systematic identification of central regulatory nodes for downstream functional interpretation.

### 4.3. Validation of CDK1 Gene

Validation of *CDK1* expression was performed using complementary bioinformatics resources. The Gene Expression Profiling Interactive Analysis (GEPIA) web server (http://gepia.cancer-pku.cn/, (accessed on 21 March 2025)) [46] was utilized to analyze mRNA expression from The Cancer Genome Atlas (TCGA, https://www.cancer.gov/ccg/research/genome-sequencing/tcga, (accessed on 21 March 2025)), investigating the distribution of *CDK1* expression in the BodyMap and its correlation with tumor stages and survival. Comparative analysis of *CDK1* expression levels in normal, tumor, and metastatic tissues was performed using the TNMplot database [47]. Parallel analyses were conducted using TNMplot, Kaplan–Meier Plotter, Human Protein Atlas, and cBioPortal to ensure cross-platform reproducibility of transcriptomic, proteomic, and genomic features. The optimal expression cutoff for *CDK1* was defined using the Kaplan–Meier Plotter’s iterative algorithm, which selects the threshold yielding the highest hazard ratio and lowest *p*-value within the interquartile expression range. Protein expression data were extracted from the Human Protein Atlas database (https://www.proteinatlas.org/, (accessed on 21 March 2025)), while genetic alteration information was accessed through cBioPortal for Cancer Genomics [48].

Protein expression patterns of CDK1 were retrieved from the Human Protein Atlas (HPA) database using the validated antibody HPA003387. This rabbit monoclonal antibody was applied at a 1:200 dilution to formalin-fixed human tissues, where CDK1 expression was visualized using 3,3’-diaminobenzidine (DAB) staining. The HPA platform utilizes a semi-automated algorithm to classify staining results into four expression categories (high, medium, low, or not detected), based on both DAB staining intensity and the percentage of positively stained cells. These standardized evaluations enhance inter-sample comparability and reproducibility across datasets. Microscopy images were acquired at 40× magnification across selected human cell lines [49], including A-431 (epidermoid carcinoma), U-251MG (glioblastoma), and U2OS (osteosarcoma), to assess the subcellular localization of CDK1. Protein expression scores and images were obtained from HPA’s publicly available Immunofluorescence repository, which is based on validated antibody protocols and centralized scoring standards.

### 4.4. Machine Learning in CDK1 Gene Expression

To further evaluate the biological relevance of *CDK1*, supervised machine learning models were employed to prioritize differentially expressed genes (DEGs) and to assess their predictive value. A Random Forest (RF) classifier was applied to rank DEGs according to Gini importance scores, while a feed-forward Artificial Neural Network (ANN) was trained to test whether these genes could reliably distinguish epithelial ovarian cancer from normal ovarian tissue. Data were normalized and partitioned using stratified 5-fold cross-validation to avoid overfitting and ensure generalizability. Both models consistently highlighted *CDK1* among the top features, with the final ANN achieving an average accuracy of 91.2% and an area under the ROC curve (AUC) of 0.94. To strengthen interpretability, recursive feature elimination and dropout regularization were integrated, and functional protein–protein interaction (PPI) networks were reconstructed using NetworkX 2.3 (NetworkX Developers, Python Software Foundation, Wilmington, DE, USA), Matplotlib 3.9 (Matplotlib Development Team, Python Software Foundation, Wilmington, DE, USA) in Python 3.11 (Python Software Foundation, Wilmington, DE, USA) and Cytoscape 3.10.0 (Cytoscape Consortium, San Diego, CA, USA) [50]. These steps provided a coherent framework linking *CDK1* expression with its regulatory partners and underscored its central role as a druggable hub in ovarian cancer.

### 4.5. Pharmacological Effects In Silico

Based on hub gene expression analysis, *CDK1* was selected as a primary target for pharmacological intervention. The protein structures of CDK1 and WEE1 were obtained from the RCSB Protein Data Bank (https://www.rcsb.org/, (accessed on 27 March 2025)) [51,52] after identification in the UniProt database (https://www.uniprot.org/, (accessed on 27 March 2025)). Protein preparation for molecular docking included removal of pre-existing ligands and extraneous water molecules, hydrogen atom addition, elimination of redundant residues, amino acid charge optimization, and energy minimization using Chimera software 1.17.2 (University of California, San Francisco, CA, USA) [53,54].

Binding sites for drug targeting were identified through the COACH database (https://zhanggroup.org/COACH/, (accessed on 27 March 2025)) [55] and supplemented with information from the PDB literature. Chemical structures of seven candidate drugs were extracted from the PubChem database (https://pubchem.ncbi.nlm.nih.gov/, (accessed on 27 March 2025)). Two-dimensional structures were defined using IUPAC nomenclature and transformed into energy-minimized three-dimensional conformations using ChemBio3D software 12.0 (PerkinElmer Informatics, Inc., Waltham, MA, USA) [56].

Molecular docking simulations were conducted using AutoDock Vina 1.2.x (The Scripps Research Institute, La Jolla, CA, USA) [57] in conjunction with PyRX 1.0 (The Scripps Research Institute, La Jolla, CA, USA) [58]. High-precision selection criteria were employed to identify ligands with optimal binding profiles. Pharmacokinetic properties were analyzed using the Protein-Ligand Interaction Profiler (PLIP, https://plip-tool.biotec.tu-dresden.de/plip-web/plip/index, (accessed on 27 March 2025)) [59] and AdmetSAR 2.0 (https://lmmd.ecust.edu.cn/admetsar2, (accessed on 27 March 2025)) [60] databases.

Molecular dynamics simulations were performed using GROMACS for 100 nanoseconds to evaluate CDK1 stability in both apo and Naringin-bound states. The CHARMM36 force field was applied, and the system was solvated in a TIP3P water molecule cubic box. Following energy minimization and equilibration in NVT and NPT ensembles, production runs were conducted with calculations of root-mean-square deviation (RMSD), radius of gyration (Rg), and root-mean-square fluctuation (RMSF) to assess conformational stability and flexibility [61].

### 4.6. Statistical Analysis

Statistical analyses were performed using R 4.3.2 (R Core Team, Vienna, Austria) and Python 3.11. Differential expression was evaluated with the limma package 3.21 in R 4.3.2, applying the Benjamini–Hochberg procedure for multiple testing correction. Network topology and enrichment analyses were conducted using igraph 2.1.4 (igraph Development Team, Vienna, Austria) and clusterProfiler 3.21 (Bioconductor project, Guangzhou Medical University, Guangzhou, China), whereas machine learning–based feature prioritization was implemented with scikit-learn. Survival correlations were analyzed with the survival package, and data visualization was carried out using ggplot2 in R 4.3.2 and matplotlib 3.9 in Python 3.11. Statistical significance was considered at *p* < 0.05, with adjusted *p*-values (FDR < 0.05) reported where applicable [62,63].

## 5. Conclusions

Transcriptome profiling combined with machine learning inference and in silico simulations identified *CDK1* as a critical regulatory hub in epithelial ovarian cancer. Dual inhibition of CDK1 and its upstream modulator WEE1 by Naringin exhibited favorable binding dynamics, structural stability, and a low predicted toxicity profile. Our findings support a novel dual-inhibition paradigm in ovarian cancer, leveraging natural compounds for multi-pathway disruption. These results position Naringin as a compelling candidate for targeted therapeutic intervention, offering a mechanistically informed approach to disrupt aberrant cell cycle progression in ovarian malignancies.

## 6. Limitations and Future Directions

While this computational investigation establishes CDK1 as a pivotal therapeutic target in epithelial ovarian cancer and demonstrates Naringin’s potential as a dual CDK1/WEE1 inhibitor, several methodological constraints warrant careful consideration. The absence of experimental validation represents the most significant limitation, as comprehensive in vitro assays examining antiproliferative mechanisms and downstream signaling pathways remain essential prerequisites for validating computational predictions. Subsequent in vivo studies will be critical for establishing therapeutic efficacy, pharmacokinetic profiles, and safety margins, given that computational frameworks cannot fully recapitulate the complex tumor microenvironment and host-drug interactions that ultimately determine clinical success. The reliance on publicly available GEO datasets, though providing valuable transcriptomic insights, introduces inherent constraints related to sample composition, particularly the markedly limited normal ovarian tissue representation across datasets (2–5 samples per cohort), potentially compromising statistical power and the reliability of differential gene expression analyses. Although empirical Bayes moderation within the LIMMA framework partially addresses small sample limitations by improving variance estimation, the fundamental issue of limited control samples persists, necessitating larger, well-characterized normal tissue cohorts to enhance statistical robustness and ensure more reliable biomarker identification.

The therapeutic landscape for ovarian cancer increasingly emphasizes combination strategies, suggesting that Naringin’s clinical potential may be best realized through rational drug combinations with established platinum-based regimens, PARP inhibitors, or emerging immunotherapeutic approaches to unlock enhanced therapeutic efficacy while potentially mitigating resistance mechanisms. Naringin’s suboptimal predicted oral bioavailability presents a significant translational challenge that could be addressed through advanced pharmaceutical approaches, including nanoformulation strategies, prodrug development, or targeted delivery systems to bridge the substantial gap between computational promise and clinical implementation. The integration of comprehensive molecular profiling platforms offers opportunities for precision medicine approaches, enabling treatment stratification based on tumor-specific genetic and epigenetic signatures. The transition from computational modeling to clinical application demands a systematic translational research program encompassing rigorous preclinical validation, optimized pharmaceutical development, and carefully designed clinical trials—coordinated efforts that will be essential for realizing Naringin’s therapeutic potential in the challenging landscape of ovarian cancer treatment.

## Figures and Tables

**Table 1:** Binding Affinities and Interaction Profiles of Candidate Compounds with CDK1 Protein.

	Prediction	CDK1 (4y72)					WEE1 (8bju)				
Drug		Binding Affinity (kcal/mol)	Binding Site	Hydrophobic Interactions	Hydrogen Bonds	Salt Bridges	Binding Affinity (kcal/mol)	Binding Site	Hydrophobic Interactions	Hydrogen Bonds	Salt Bridges
Adavosertib		*	*	*	*	*	−11	ASN376/CYS379	8	4	1
Alsterpaullone		−10.9	TYR 15/ASP 86	8	0	0	−10.4	CYS379	8	2	0
Avotaciclib		−9.3	TYR 15/GLN 132	5	3	1	−8.7	ASN376/CYS379	4	5	0
Fostamatinib		−12.5	TYR 15	7	6	0	−7.5	ASN376	1	4	2
Olomoucine		−8.5	TYR 15/GLN 132	6	4	1	*	*	*	*	*
Seliciclib		−8.7	TYR 15/GLN 132	10	4	0	*	*	*	*	*
Naringin		−10.6	TYR 15/GLN 132	6	8	0	−9.6	ASN376/CYS379	5	5	1

* The asterisk indicates a lack of binding at the identified binding site in the target protein.

**Table 2:** Predicted Pharmacokinetic and Toxicological Properties of Selected CDK1 Inhibitors.

Name of the Drug		Admet SAR						
	Subcellular Localization	AlogP	Molecular Weight	Blood–Brain Barrier	Human Oral Bioavailability	Nephrotoxicity	Hepatotoxicity	Ames Mutagenesis
Adavosertib	Mitochondria	2.89	500.607	+	0.6429(+)	0.7773(−)	0.7075(+)	0.54(−)
Alsterpaullone	Mitochondria	3.24	293.276	+	0.7143(+)	0.5739(+)	0.7875(+)	0.88(+)
Avotaciclib	Mitochondria	0.87	281.279	+	0.5571(+)	0.4864(+)	0.6125(+)	0.5(−)
Fostamatinib	Mitochondria	3.09	580.459	−	0.5571(+)	0.7326(+)	0.6677(+)	0.53(−)
Olomoucine	Nucleus	1.36	298.343	+	0.5714(−)	0.5939(−)	0.5587(+)	0.58(+)
Seliciclib	Lysosomes	3.2	354.449	+	0.5143(+)	0.7729(−)	0.5538(−)	0.59(−)
Naringin	Mitochondria	−1.17	580.54	−	0.9857(−)	0.6977(−)	0.8750(−)	0.61(−)

## Data Availability

The datasets generated during and/or analyzed during the current study are available in the [GSE DataSets, TCGA, and Human Protein Atlas] repository, [https://www.ncbi.nlm.nih.gov/geo/query/acc.cgi?acc=GSE2879, (accessed on 21 March 2025), https://www.ncbi.nlm.nih.gov/geo/query/acc.cgi?acc=GSE14407, (accessed on 21 March 2025), https://www.ncbi.nlm.nih.gov/geo/query/acc.cgi?acc=GSE54388, (accessed on 21 March 2025), https://www.ncbi.nlm.nih.gov/geo/query/acc.cgi?acc=GSE30219, (accessed on 21 March 2025), https://www.ncbi.nlm.nih.gov/geo/query/acc.cgi?acc=GSE33532, (accessed on 21 March 2025), https://www.ncbi.nlm.nih.gov/geo/query/acc.cgi?acc=GSE19188, (accessed on 21 March 2025)], [https://toil.xenahubs.net/download/TcgaTargetGtex_rsem_gene_tpm.gz, (accessed on 21 March 2025)], [https://toil.xenahubs.net/download/TcgaTargetGTEX_phenotype.txt.gz, (accessed on 21 March 2025)] and [https://www.proteinatlas.org/accession numbers, ENSG00000170312, (accessed on 21 March 2025)].

## Supplementary Materials

The supporting information can be downloaded at https://www.mdpi.com/article/10.3390/ijms26189168/s1.
