# Supplementary material for: Integrated Analysis, Machine Learning, Molecular Docking and Dynamics of CDK1 Inhibitors in Epithelial Ovarian Cancer: A Multifaceted Approach Towards Targeted Therapy
Source: Int J Mol Sci. 2025 Sep 19;26(18):9168. doi: 10.3390/ijms26189168 (PMC12470616; doi:10.3390/ijms26189168)
Supplement: Supplementary file 1 [file ijms-26-09168-s001.zip › ijms-3783363-supplementary.pdf]

Integrated Analysis, Machine Learning, Molecular Docking and Dynamics of CDK1 Inhibitors in Epithelial Ovarian Cancer: A Multifaceted Approach Towards Targeted Therapy

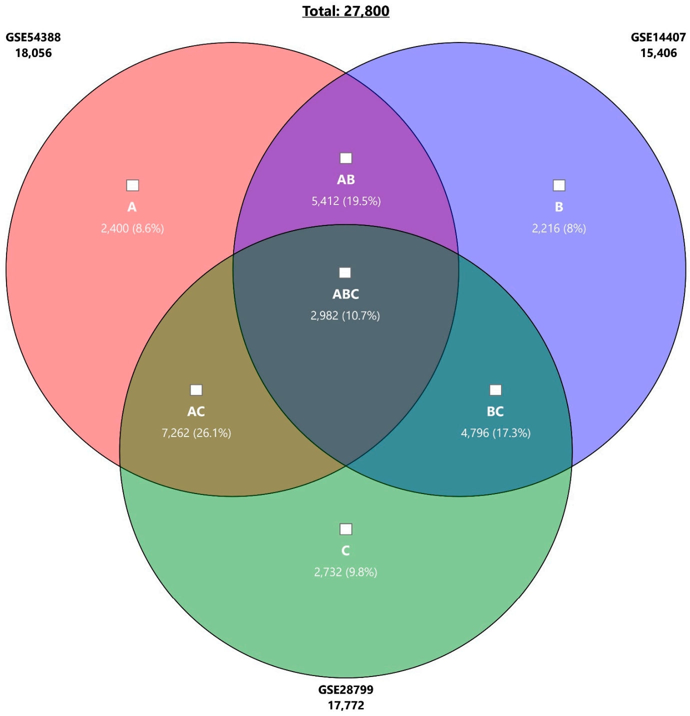

Figure S1. Venn diagram showing the overlap among the three gene expression datasets.

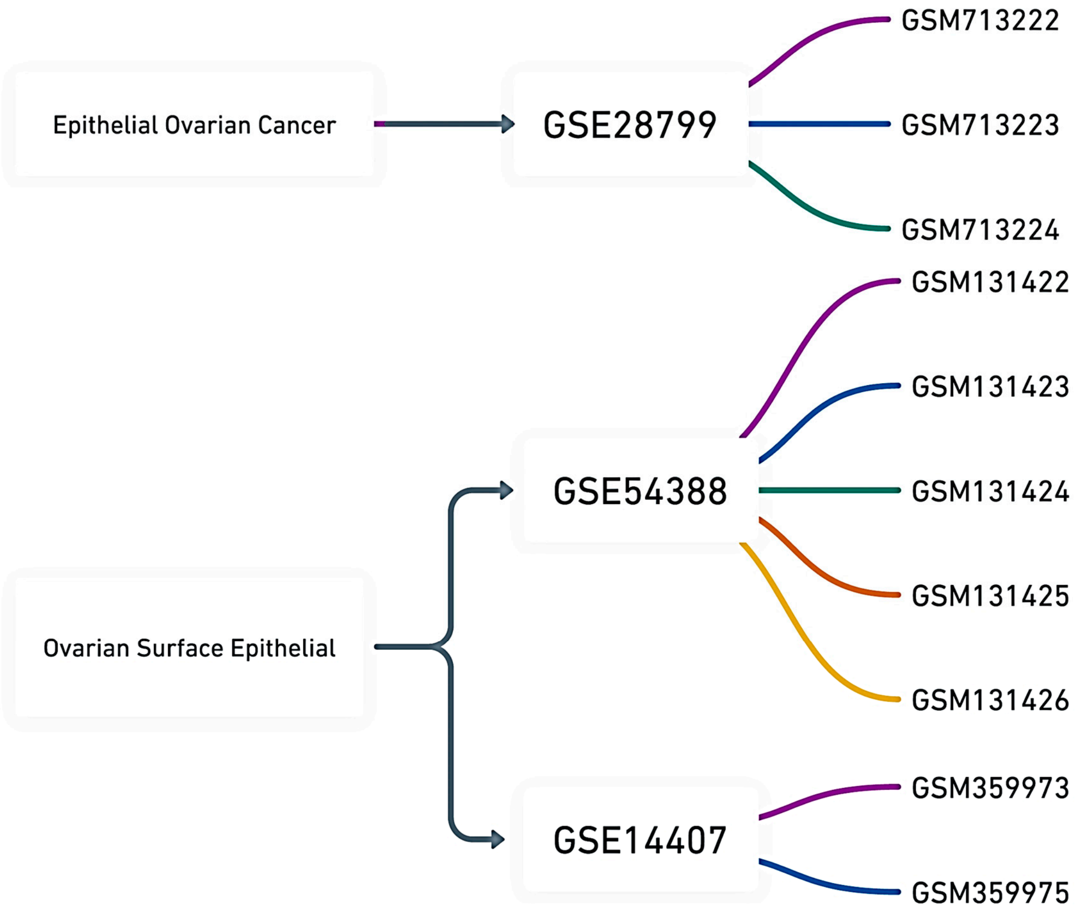

Figure S2. Mapping of GSM identifiers to their corresponding GSE datasets, enabling clear identification of sample origin.

Table S1. The results of 2D and 3D molecular docking interactions between the proteins CDK1 and WEE1 and seven potential drugs

**Protein-Ligand**	3D	2D	3D
**CDK1-Adavosertib**	*	*	*
**CDK1-Alsterpaullone**	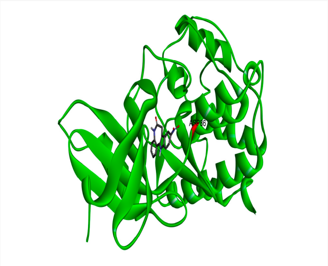	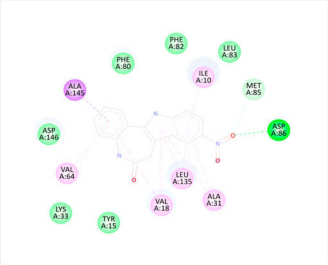	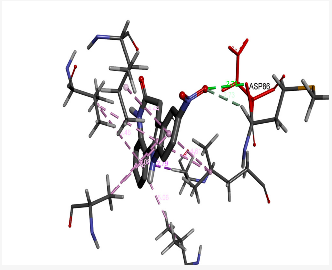
**CDK1-Avotaciclib**	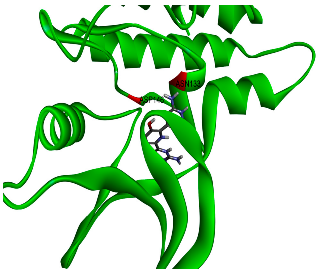	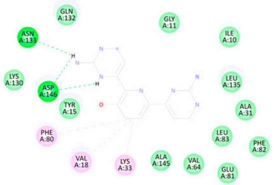	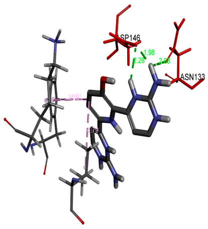
**CDK1-Fostamatinib**	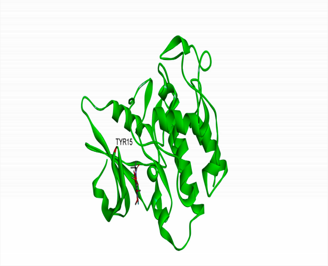	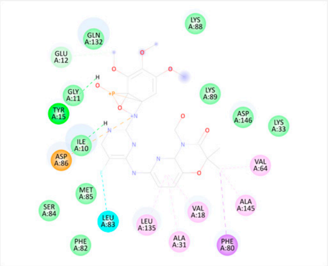	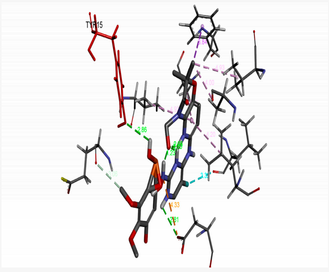
**CDK1-Olomoucine**	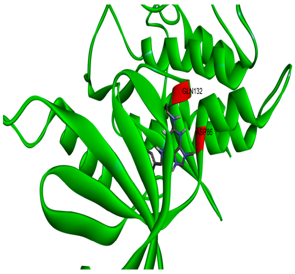	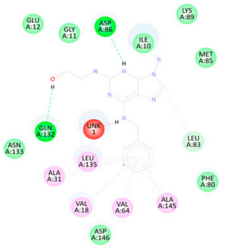	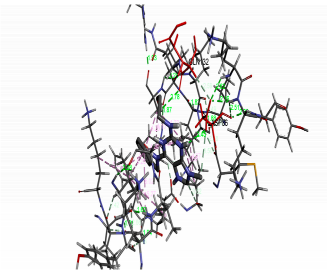
**CDK1-Seliciclib**	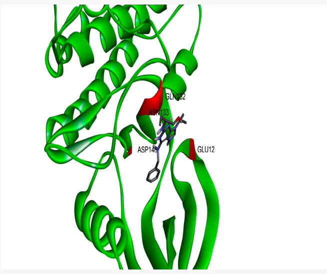	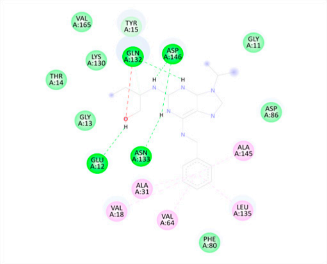	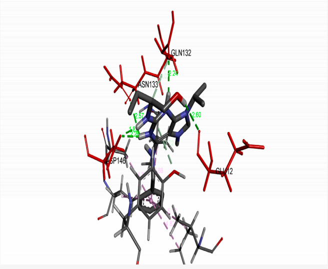

Continue Supplementary table

Protein-Ligand	3D	2D	3D
**CDK1-Naringin**	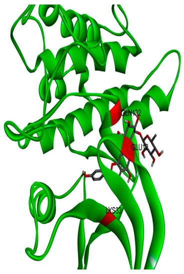	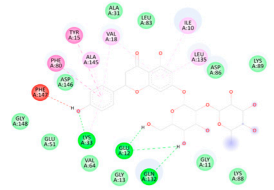	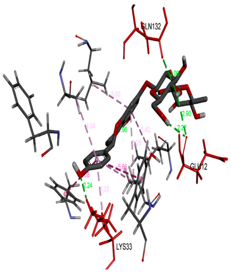
**WEE1-Adavosertib**	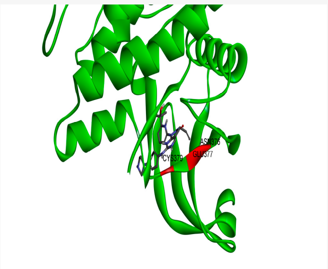	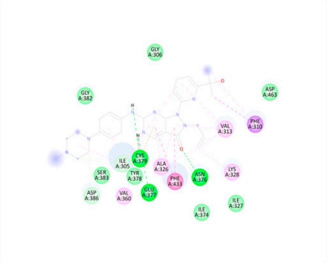	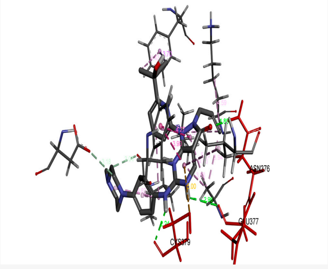
**WEE1-Alsterpaullone**	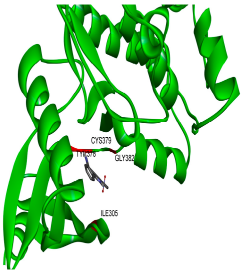	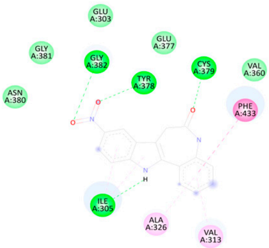	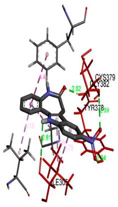
**WEE1-Avotaciclib**	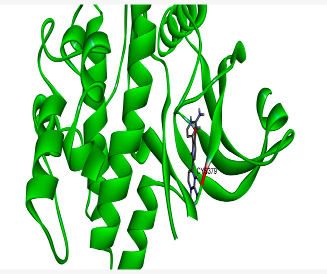	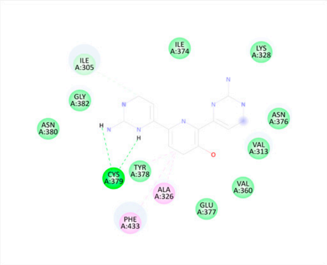	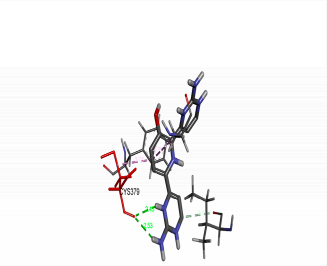
**WEE1-Fostamatinib**	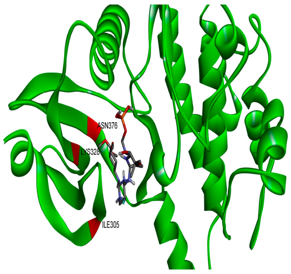	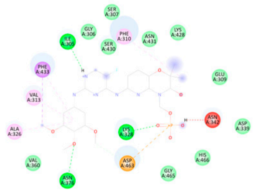	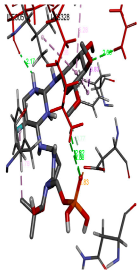
**WEE1-Olomoucine**	*	*	*
**WEE1-Seliciclib**	*	*	*
**WEE1-Naringin**	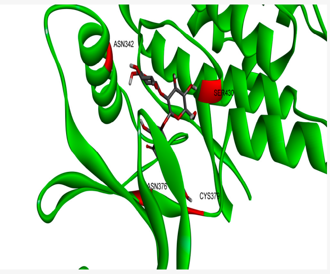	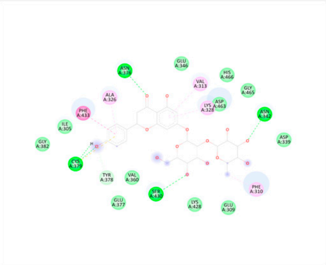	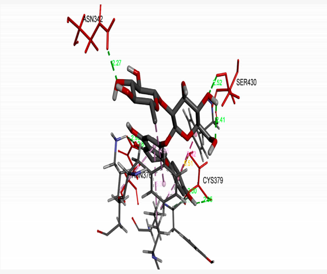

TABLE S2. LIST OF CANCER TYPES ANALYZED IN THIS STUDY, INCLUDING THEIR STANDARD TCGA ABBREVIATIONS AND FULL CANCER NAMES.

ABBREVIATION	Full Cancer Name
**LAML**	Acute Myeloid Leukemia
**LGG**	Brain Lower Grade Glioma
**GBM**	Glioblastoma Multiforme
**TGCT**	Testicular Germ Cell Tumors
**THYM**	Thymoma
**DLBC**	Diffuse Large B-cell Lymphoma
**PCPG**	Pheochromocytoma and Paraganglioma
**KICH**	Kidney Chromophobe
**SKCM**	Skin Cutaneous Melanoma
**HNSC**	Head and Neck Squamous Cell Carcinoma
**ESCA**	Esophageal Carcinoma
**THCA**	Thyroid Carcinoma
**STAD**	Stomach Adenocarcinoma
**OV**	Ovarian Serous Cystadenocarcinoma
**READ**	Rectum Adenocarcinoma
**COAD**	Colon Adenocarcinoma
**CESC**	Cervical Squamous Cell Carcinoma and Endocervical Adenocarcinoma
**LIHC**	Liver Hepatocellular Carcinoma
**PRAD**	Prostate Adenocarcinoma
**LUSC**	Lung Squamous Cell Carcinoma
**LUAD**	Lung Adenocarcinoma
**BRCA**	Breast Invasive Carcinoma
**UCS**	Uterine Carcinosarcoma
**UCEC**	Uterine Corpus Endometrial Carcinoma
**BLCA**	Bladder Urothelial Carcinoma
**CHOL**	Cholangiocarcinoma
**PAAD**	Pancreatic Adenocarcinoma
**SARC**	Sarcoma
**KIRP**	Kidney Renal Papillary Cell Carcinoma
**KIRC**	Kidney Renal Clear Cell Carcinoma
**ACC**	Adrenocortical Carcinoma
